# ^13^C-labeling reveals non-conventional pathways providing carbon for hydroxy fatty acid synthesis in *Physaria fendleri*

**DOI:** 10.1093/jxb/erad343

**Published:** 2023-09-05

**Authors:** Jean-Christophe Cocuron, Ana Paula Alonso

**Affiliations:** BioAnalytical Facility, University of North Texas, Denton, TX 76203, USA; BioAnalytical Facility, University of North Texas, Denton, TX 76203, USA; BioDiscovery Institute and Department of Biological Sciences, University of North Texas, Denton, TX 76203, USA; University of Nebraska-Lincoln, USA

**Keywords:** ^13^C-labeling, alternative crops, carbon conversion efficiency, hydroxy fatty acids, isocitrate dehydrogenase, malic enzyme, oilseed, *Physaria fendleri*, plant metabolism, Rubisco

## Abstract

*Physaria fendleri* is a member of the *Brassicaceae* that produces in its embryos hydroxy fatty acids, constituents of oils that are very valuable and widely used by industry for cosmetics, lubricants, biofuels, etc. Free of toxins and rich in hydroxy fatty acids, *Physaria* provides a promising alternative to imported castor oil and is on the verge of being commercialized. This study aims to identify important biochemical step(s) for oil synthesis in *Physaria*, which may serve as target(s) for future crop improvement. To advance towards this goal, the endosperm composition was analysed by LC-MS/MS to develop and validate culture conditions that mimic the development of the embryos *in planta*. Using developing *Physaria* embryos in culture and ^13^C-labeling, our studies revealed that: (i) *Physaria* embryos metabolize carbon into biomass with an efficiency significantly lower than other photosynthetic embryos; (ii) the plastidic malic enzyme provides 42% of the pyruvate used for *de novo* fatty acid synthesis, which is the highest measured so far in developing ‘green’ oilseed embryos; and (iii) *Physaria* uses non-conventional pathways to channel carbon into oil, namely the Rubisco shunt, which fixes CO_2_ released in the plastid, and the reversibility of isocitrate dehydrogenase, which provides additional carbon for fatty acid elongation.

## Teaser

Plastidic malic enzyme provides considerable carbon and reductant for fatty acid synthesis in *Physaria* embryos. Non-conventional pathways, namely the Rubiscoshunt and isocitrate dehydrogenase reversibility, fix CO_2_ released by other reactions.

## Introduction

Developing alternative sources of domestic energy is becoming essential, not only to replace non-renewable fossil fuels and petroleum-based chemicals, but to alleviate environmental, strategic, and economic impacts too. One strategy to address those issues is to invest in alternative crops that can grow off-season or on marginal land, and produce compounds to substitute for petroleum-based fuels and chemicals. *Physaria fendleri* has been identified as a promising alternative crop. *Physaria* seeds produce approximately 25% oil (w/w) of which 60% is (11*Z*,14*R*)-14-hydroxyicos-11-enoic acid (aka lesquerolic acid) (Barclay *et al*., 1962; Isbell *et al.*, 2008), a highly valued hydroxy fatty acid (HFA) (Cocuron *et al.*, 2014). This special class of fatty acids (FAs) is used industrially in the manufacture of lubricant greases, cosmetics, coatings, paints, biofuels, etc. The current source of HFAs is imported castor (*Ricinus communis*), which produces ricinoleic acid (Cocuron *et al.*, 2014). However, castor plants are also a source of highly toxic compounds (ricin and ricinin) and cause strong allergic reactions, preventing the production of castor oil domestically. On the other hand, *Physaria*, a native plant from the southwest of the USA, is free of toxins, which makes it a promising alternative source of HFAs to imported castor oil. *Physaria* is a winter crop, meaning that this perennial plant can be placed in rotation with commodity crops such as corn and soybean. The current seed yields are approximately 2000 kg ha^−1^ (Isbell, 2009) but can potentially reach 2500–3000 kg ha^−1^ (Dierig *et al.*, 2011). It is important to note that *Physaria* can be used as an off-season cover crop, which has the tremendous advantage of preventing soil erosion and nutrient loss. However, for this plant to become an economically viable source of HFAs, molecular and biochemical resources need to be developed to enhance oil production by breeding and/or genetic manipulation.

To date, tentative efforts to engineer other related *Brassicaceae* species (Arabidopsis or *Camelina*) with the castor or *Physaria* gene *FAH12* (FA hydroxylase 12) have had limited success: not only have HFAs only reached 30% (w/w FA), but a drop in total seed oil content was often reported too (van de Loo *et al.*, 1995; Broun *et al.*, 1998; Moon *et al.*, 2001; Smith *et al.*, 2003; Kumar *et al.*, 2006; Lu *et al.*, 2006; Dauk *et al.*, 2007; Burgal *et al.*, 2008; Lu and Kang, 2008; Kim *et al.*, 2011; van Erp *et al.*, 2011; Hu *et al.*, 2012; Wayne and Browse, 2013; Snapp *et al.*, 2014; Bayon *et al.*, 2015). More recent engineering of Arabidopsis, stacking five genes in a *fae1* mutant background, reached up to 37% HFAs with 15% decrease in total oil content (Shockey *et al.*, 2019) and 34% HFAs without impacting oil accumulation (Lunn *et al.*, 2019, 2022). In order to understand the limitation in engineering other species to produce HFAs, a transcriptomic study was conducted, comparing *Physaria*, wild-type *Camelina*, and *Camelina* expressing the castor gene *FAH12* (Horn *et al.*, 2016). This study revealed that adaptations occurred for at least 20 genes involved in fatty acid synthesis (FAS), regulation, and triacyl glycerol assembly. Indeed, *Physaria* lipid genes appear to have co-evolved through both modulation of transcriptional abundance and alteration in protein sequence. These results indicate that the insertion of at least 20 genes might be necessary to engineer a related species to produce HFAs at similar levels to *Physaria*, which would be extremely challenging. The alternative approach would be to understand HFA biosynthesis pathways and improve those directly in *Physaria*.

In oilseeds, *de novo* FAS of acyl chains, up to 18 carbons, occurs in the plastids of the embryos and requires carbon (acetyl-CoA), energy (ATP), and reducing power (NADPH and NADH) (Hills, 2004; Sagun *et al.*, 2023). Sucrose, which usually serves as a carbon source, is transported into the embryo and cleaved to generate hexose phosphates, which are metabolized through glycolysis and the oxidative pentose-phosphate pathway (OPPP), producing carbon precursors for FAS in the form of acetyl-CoA. It is important to note that acetyl-CoA cannot cross the plastid membrane (Weaire and Kekwick, 1975; Roughan *et al.*, 1979). Hence, precursors for acetyl-CoA synthesis must be generated in the plastid or imported from the cytosol. Studies on isolated plastids have demonstrated that a broad range of cytosolic metabolites such as glucose 6-phosphate (glucose 6-P), phospho*enol*pyruvate (PEP), pyruvate, and malate are capable of supporting FAS (Smith *et al.*, 1992; Kang and Rawsthorne, 1994, 1996; Qi *et al.*, 1995; Rawsthorne and Eastmond, 2000; Pleite *et al.*, 2005; Sagun *et al.*, 2023). These can be taken up and utilized for oil production at different rates by plastids from various plant tissues, species, and stages of development. FAS also depends upon supplies of ATP and reducing power. In green seeds, light energy can be used by chloroplasts to generate ATP and NADPH (Browse and Slack, 1985; Ohlrogge *et al.*, 2004; Schwender *et al.*, 2004, 2006; Goffman *et al.*, 2005; Allen *et al.*, 2009; Hay *et al.*, 2014; Tsogtbaatar *et al.*, 2015; Acket *et al.*, 2020; Carey *et al.*, 2020), whereas plastids from heterotrophic tissues must either generate these compounds internally or import them from the cytosol (Browse and Slack, 1985; Hill and Smith, 1991; Kleppinger-Sparace *et al*., 1992; Smith *et al.*, 1992; Neuhaus *et al.*, 1993; Kang and Rawsthorne, 1996; Alonso *et al.*, 2007, 2010a, 2011; Cocuron *et al.*, 2019b). Besides photosynthesis, the main metabolic pathways generating ATP and NADPH are mitochondrial respiration and the OPPP, respectively. For oilseeds producing longer FAs, such as *Physaria* accumulating lesquerolic acid, FA elongation occurs in the endoplasmic reticulum and requires acetyl-CoA, energy, and reducing power. The acetyl-CoA, used as a building block for FA elongation, is formed in the cytosol from citrate by citrate lyase (Sagun *et al.*, 2023).

To understand the metabolic pathways that are active during FAS in developing *Physaria* embryos, a metabolomics study was previously conducted, showing that: (i) glucose and glutamine are the major sources of carbon and nitrogen for *Physaria* embryos during FAS; (ii) malate and citrate are the main organic acids present in developing *Physaria* embryos, suggesting their respective contribution as sources of carbon skeletons for FAS and elongation; (iii) the presence of ribulose 1,5-bisphosphate (bisP) across the developmental stages provides evidence for Calvin cycle activity, which indicates that part of the ATP and NADPH required for FAS is produced by photosynthetic conversion of light energy; and (iv) the OPPP and the tricarboxylic acid (TCA) cycle, which are important for providing reductants and energy, are present in developing *Physaria* embryos (Cocuron *et al.*, 2014). The present study aims to identify important biochemical step(s) for oil synthesis in *Physaria*, which may serve as target(s) for future crop improvement. To advance towards this goal, the endosperm composition was analysed by LC-MS/MS to develop and validate culture conditions that mimic the development of the embryos *in planta*. Using developing *Physaria* embryos in culture allowed us to: (i) determine the efficiency in which embryos convert substrates into biomass components (aka carbon conversion efficiency), and (ii) replace the substrates by ^13^C-labeled ones and identify important sources of carbon and reductant for HFA synthesis in *Physaria*.

## Materials and methods

### Chemicals

Potassium hydroxide solution (45% w/w) and GC/LC-MS grade solvents and additives such as acetonitrile, *n*-hexanes, acetic acid, and formic acid were ordered from Fisher Scientific. Three molar methanolic HCl, toluene, *N*-butylamine, 1000× Gamborg’s vitamin solution, gibberellic acid, and other standards were purchased from MilliporeSigma. [U-^13^C_7_]Benzoic acid, [1,2-^13^C_2_]glucose, [U-^13^C_6_]glucose, [U-^13^C_5_]glutamine, [U-^13^C_2_]glycine, and [U-^13^C_12_]sucrose were ordered from Isotec. Murashige and Skoog (MS) basal salt was obtained from PhytoTechnology Laboratories. Ultrapure water used for the LC-MS/MS analyses was from a Milli-Q system from MilliporeSigma.

### Plant growth

*Physaria* seeds of the PI 610492 accession were ordered from the North Central Regional Plant Introduction Station. Seed germination, plant growth, and daily flower hand-pollination and tagging were performed as previously described (Cocuron *et al.*, 2014).

### Endosperm collection and analysis

Liquid endosperm from *Physaria* seeds 18–20 days after pollination (DAP) was harvested following the procedure previously published (Tsogtbaatar *et al.*, 2020). Briefly, 3–5 μl of liquid endosperm, corresponding to approximatively 100 *Physaria* seeds (18–20 DAP), was collected under a dissecting microscope using a 3/10 ml insulin syringe, and processed the same way as by Tsogtbaatar *et al.* (2020) before storage at −80 °C. Amino acids and sugars present in *Physaria* liquid endosperm were extracted using boiling water as previously described (Tsogtbaatar *et al.*, 2020). Then, these compounds were separated and quantified by LC-MS/MS following the same chromatographic and mass spectrometric conditions as previously published (Cocuron *et al.*, 2014; Tsogtbaatar *et al.*, 2015). Hormones were extracted by adding 100 μl of 10% aqueous methanol supplemented with 1% acetic acid to each tube containing 5 nmol of [U-^13^C_7_]benzoic acid. Samples were vortexed, transferred to 0.2 μm Nanosep filtering devices, and centrifuged at 17 000 *g* at 4 °C for 5 min. Eluates were diluted by 10 for LC-MS/MS analysis and analysed as previously described (Cocuron *et al.*, 2019a; Tsogtbaatar *et al.*, 2020). The classes of metabolites mentioned above were quantified using internal standards and known concentrations of external standards.

### Culture conditions for developing *Physaria* embryos

*Physaria* silicles at 18 DAP were harvested into 50 ml conical tubes seated on ice; 20 ml of 20% bleach solution was added to the tube, and silicles were sterilized for 5 min. Then, the silicles were rinsed with sterile water a total of five times under aseptic conditions. Silicles were dissected under a microscope to retrieve embryos, which were immediately transferred to a six-well tissue culture plate. Ten embryos were added to each well containing double-glass fiber filters (30 mm diameter) saturated with 1 ml of medium composed of 10 mM HEPES (pH 6.3), 40 mM glucose, 5 mM glutamine, 4.3 g l^–1^ Murashige and Skoog basal medium, 1× Gamborg’s vitamin solution, 10 μM abscisic acid, and 22.5% polyethylene glycol 4000. The six-well tissue culture plate was covered with a green cellophane film to mimic the silicle light absorption, and embryos were incubated for 9 d at 21 °C under a constant light intensity of 12 μmol m^–2^ s^–1^. At the end of the culture time, 27-DAP embryos were collected, rinsed extensively with ultrapure water to remove residual medium, flash-frozen in liquid nitrogen, lyophilized for 3 d, and kept in a −80 °C freezer until further processing.

### Biomass analysis

Biomass (lipids, proteins, starch) extraction was performed as previously published (Cocuron *et al.*, 2014). Lipids were methylated to obtain FA methyl esters that were diluted by factor of 2 before injection through the GC-MS system. Quantification of FA methyl esters, total proteins, and starch was carried out using previously published methods (Cocuron *et al.*, 2014; Tsogtbaatar *et al.*, 2015). Quantification of the cell wall was obtained by subtracting the biomass components mentioned above from the total biomass. Acidic hydrolysis using 6 M HCl of 250 μl of total protein extract was conducted to determine the composition of amino acids (McClure *et al.*, 2017). Analysis of proteinogenic amino acids was done using LC-MS/MS as previously described (McClure *et al.*, 2017).

### Carbon conversion efficiency determination

Carbon conversion efficiency (CCE) of developing *Physaria* embryos was determined as previously published (Tsogtbaatar *et al.*, 2020). CCE is defined as follows, where CCE is a percentage and total carbon is in µmoles per embryo:


$$\begin{array}{l} CCE\;\left(\%\right)=\frac{total\;carbon\;into\;biomass}{total\;carbon\;uptake}\times 100 \end{array}$$ (1)

### Total carbon uptake

To estimate carbon uptake, the initial amounts of substrates were compared with the remaining quantities in the medium after incubating 18-DAP *Physaria* embryos, as described earlier. Additionally, control culture plates containing only the medium (without embryos) were set up in parallel. After 9 d of incubation, the embryos were collected, and each well, including those with only the medium, received 1 ml of a standard mixture consisting of 20 mM [U-^13^C_6_]glucose and 30 mM [U-^13^C_2_]glycine. Then, the medium samples were processed exactly the same way as in Tsogtbaatar *et al.* (2020) regarding the preparation, analysis through the LC-MS/MS, and the determination of quantities of glucose and glutamine. The consumption of glucose (Glc) and glutamine (Gln) was assessed by calculating the difference between their initial concentrations and the concentrations remaining after the experiment. Subsequently, these substrate uptake values were utilized to determine the total carbon uptake (µmol per embryo) and then expressed in units of nmol embryo^−1^ h^−1^:


$$\begin{array}{l} Total\;C\;uptake\;=Gln\;consumed\;\times \;5\;+\;Glc\;consumed\;\times \;6 \end{array}$$ (2)

### Carbon into biomass

Each biomass component underwent the following calculations: (i) the accumulated amount in grams over a 9 d culture period (from 18 to 27 DAP); (ii) the average molecular weight; (iii) the number of moles per embryo (measured in µmol per embryo); and (iv) the total carbon number. The conversion of total carbon into biomass (oil, protein, and carbohydrate) for each component is given by:


$$\begin{array}{lll} Total\;C\;into\;biomass= & \sum (each\;biomass\; & \;component\times \;total\;C\;in\;the\;component)\; \end{array}$$ (3)

The carbon content in each biomass component was determined using Equations 4–6. To calculate the total carbon number (µmol C per embryo) converted into oil, the following procedure was employed:


$$\begin{array}{l} \begin{array}{lllll} & \;\mu moles\;C\;per\;embryo=\; & \sum \left(\frac{abundance\;of\;each\;FA}{MW\;of\;each\;FA}\right)\;\times \frac{10^{6}}{n}\;\; & \times \sum (fraction\;of\;each\;FA\;in\;oil\;\; & \times \;carbon\;number\;of\;the\;respective\;FA\;molecule)\; \end{array} \end{array}$$ (4)

where *n* is number of embryos, MW is molecular weight in g mol^−1^, and FA is fatty acid.

To determine the total carbon content in proteins, the quantification of each amino acid (AA) from storage proteins was determined in grams based on the amino acid composition obtained from hydrolysed proteins. It is worth mentioning that the molecular weight of each AA was calculated with consideration of water loss (18 g mol^–1^). Ultimately, the total carbon number (µmol C per embryo) converted into proteins was calculated using the following method:


$$\begin{array}{l} \begin{array}{lllll} & \;\mu moles\;C\;per\;embryo\; & =\sum \left(\frac{abundance\;of\;each\;AA}{MW\;of\;each\;AA}\right)\times \frac{10^{6}}{n}\; & \times \sum (fraction\;of\;each\;AA\;in\;protein\;\; & \times \;carbon\;number\;of\;each\;AA\;molecule)\; \end{array} \end{array}$$ (5)

To calculate the total carbon content in carbohydrates, the molecular weights and total carbon numbers for starch and cell wall were assumed to be equivalent to those of the glucose monomer. Consequently, the values used for the molecular weight and total carbon number were 162 g mol^−1^ (accounting for water loss during polymerization) and 6, respectively:


$$\begin{array}{l} \;\mu moles\;C\;per\;embryo\;=\sum \left(\frac{abundance\;of\;each\;carbohydrate}{{162\;g\;mol}^{-1}}\right)\times \frac{10^{6}}{n}\times \;6 \end{array}$$ (6)

### ^13^C-labeling of *Physaria* embryos in culture

^13^C-labeling of *Physaria* embryos in culture was designed as previously published (Tsogtbaatar *et al.*, 2020). Briefly, a medium made of a 20% [U-^13^C_5_]glutamine and 20% [U-^13^C_6_]glucose mixture was used to determine whether or not *Physaria* embryos were at metabolic isotopic steady state. Then, a second set of ^13^C-labeling experiments were performed on *Physaria* embryos to precisely cover central metabolism, consisting of (i) 20% [U-^13^C_6_]glucose and 80% [1,2-^13^C_2_]glucose and 100% ^12^C-glutamine, and (ii) 100% ^12^C-glucose and 100% [U-^13^C_5_]glutamine. Four biological replicates were utilized for each labeling experiment.

### Extraction of ^13^C-labeled oil, starch, and ^13^C-labeled polar compounds

#### ^13^C-biomass

^13^C-labeled biomass from ten 27-DAP *Physaria* embryos was sequentially extracted as previously described (Tsogtbaatar *et al.*, 2015). ^13^C-oil and ^13^C-starch extracts (*n*=4 biological replicates) were stored in a −20 °C freezer until further analysis.

### ^13^C-polar metabolites

^13^C-labeled polar metabolites (sugars, amino acids, organic acids, phosphorylated compounds) from ten 27-DAP *Physaria* embryos were extracted with boiling water, and lyophilized as previously published (Alonso *et al.*, 2010b; Koubaa *et al.*, 2013; Cocuron and Alonso, 2014; Cocuron *et al.*, 2017). Freeze-dried extracts (*n*=4 biological replicates) were stored in a −20 °C freezer until LC-MS/MS analysis.

### Quantification of ^13^C-labeling in ^13^C-lipids, ^13^C-starch, and ^13^C-polar compounds

#### Analysis of ^13^C-oil by GC-MS

^13^C-oil was derivatized into fatty acid butylamides (Allen *et al.*, 2007) and analysed by GC-MS as previously reported (Tsogtbaatar *et al.*, 2020). Note that the holding time at the end of the GC-MS run was 10 min instead of 6 min in order to have butylated lesquerolic acid eluting from the capillary column. The retention time of fatty acid butylamide derivatives was determined by obtaining total ion chromatograms in the mass range of 40–450 amu, using a scan time of 71 ms. Then, selective ion monitoring was employed to specifically track two sets of ions: (i) molecular ions at 311 and 381 amu, corresponding to butyl amide derivatives of palmitic (16C) and lesquerolic (20C) acids, respectively, and (ii) ions at 115, 116, and 117 amu, representing the *M*+0, *M*+1, and *M*+2 mass isotopomers of the McLafferty fragments resulting from the butyl amide derivatization of palmitic and lesquerolic acids. Ultimately, C1–2 from 16C and 20C fatty acids can be compared to determine the labeling of plastidic acetyl-CoA and cytosolic acetyl-CoA, respectively.

#### Analysis of ^13^C-starch by LC-MS/MS

The mass isotopomer distribution (MID) of ^13^C-glucosyl units from starch was assessed using LC-MS/MS as previously published (Tsogtbaatar *et al.*, 2020).

#### Analysis of ^13^C-labeled intracellular metabolites

Cold ultrapure water (350 μl) was added to freeze-dried extracts containing ^13^C-polar metabolites. Briefly, LC-MS/MS analysis using a multiple reaction monitoring scan survey was conducted to determine the MID of ^13^C-glucose (Cocuron *et al.*, 2020), ^13^C-free amino acids (Cocuron *et al.*, 2017), and ^13^C-organic acids and phosphorylated compounds (Alonso *et al.*, 2010b; Koubaa *et al.*, 2013; Cocuron and Alonso, 2014). In order to assess the MID of cytosolic hexose phosphates, namely glucose 6-P and fructose 6-P, ^13^C-sucrose was cleaved into ^13^C-glucose and ^13^C-fructose using an invertase as previously described (Cocuron *et al.*, 2020).

### Refixation of CO _2_ by Rubisco

The recapture of CO_2_ by Rubisco was calculated as described (Tsogtbaatar *et al.*, 2020).

Initially, the relative contributions of plastidic glycolysis and NADP-malic enzyme to the pyruvate pool were determined using:


$$\begin{array}{l} V_{glycop}+V_{mep}=1 \end{array}$$

$$\begin{array}{l} V_{glycop}\;\;\;C1\left(Phe\right)+V_{mep}\;\times \;C1\left(Met\right)=\left(V_{glycop}+V_{mep}\right)\;\times \;C1\left(Val\right) \end{array}$$ (7)

where *V*_glycop_ is the proportion of pyruvate produced by plastidic glycolysis and *V*_mep_ is the proportion of pyruvate produced by plastidic NADP-malic enzyme. The labeling abundances of C1 fragments for phenylalanine, valine, and methionine are denoted as C1(Phe), C1(Val), and C1(Met), respectively (as shown in Table 1).

**Table 1.** Use of unconventional pathways in developing oilseed embryos

Plant embryos	IDH reversibility	Plastidic PYR from NADP-malic enzyme (%)	Plastidic PGA from Rubisco (%)	References
Flax	Yes	<1	0	Acket *et al.* (2020)
Rapeseed	Yes	<1	36–64	Schwender *et al.* (2004, 2006), Hay *et al.* (2014)
*Camelina*	Yes	9	0	Carey *et al.* (2020)
Soybean	Yes	<20	14	Allen *et al.* (2009)
Pennycress	Yes	20	25	Tsogtbaatar *et al.* (2020)
*Physaria*	Yes	42	25	This study
Sunflower	No	7	0	Alonso* et al.* (2007)
Maize	No	30–54	0	Alonso* et al.* (2010a), Cocuron* et al.* (2019b)

Highlighted in grey are embryos that are not photosynthetically active. For all the studies reported above, ^13^C-labeling was conducted in developing embryos until metabolic and isotopic steady state. Labeling data for intracellular metabolites were used to determine the reversibility of the isocitrate dehydrogenase (IDH), the percentage of plastidic pyruvate (PYR) and phosphoglycerate (PGA) generated from the plastidic NADP-dependent malic enzyme (*V*_mep_) and Rubisco, respectively.

Next, the labeling enrichment of plastidic CO_2_, depicted as *F*(CO_2_), was calculated:


$$\begin{array}{l} F\;\left(CO_{2}\right)=\left(V_{glycop}+V_{mep}\right)\;\times \;C1\left(Val\right)+V_{mep}\;\times \;C1\;\left(Met\right) \end{array}$$ (8)

Lastly, the relative contribution of Rubisco to phosphoglycerate (PGA) synthesis in the plastid was determined (Schwender *et al.*, 2004):


$$\begin{array}{l} \frac{2\;F\left(C1\;of\;PGA\right)}{F\left(CO_{2}\right)}=PGA\;from\;Rubisco \end{array}$$ (9)

where *F*(C1 of PGA) and *F*(CO_2_) refer to the labeling enrichments of the C1 of PGA and CO_2_, respectively.

### Natural abundance correction for ^13^C-biomass and intracellular metabolites

The correction for the natural abundances of the isotopes of C, H, N, O, and S was performed as previously reported (Tsogtbaatar *et al.*, 2020).

### Statistical analysis

Student’s *t*-test (two tailed, type 3) was used, and *P*-values <0.05 were considered statistically significant.

## Results and discussion

### Setting up culture conditions for developing *Physaria* embryos

We have previously shown that the synthesis of HFA occurs in *Physaria* embryos, and that a linear accumulation of the biomass components (oil, protein, and starch) occurred between 18 and 33 DAP. It is important to note that the developmental stage considered in this study did not cover the maturation of the *Physaria* embryos (Cocuron *et al.*, 2014). Establishing culture conditions that mimic the development of *Physaria* embryos is key to determining their CCE and trace the main pathways involved in oil biosynthesis using ^13^C-labeling. Culture conditions have been successfully established for other *Brassicaceae* embryos (*Camelina*, canola, Arabidopsis, and pennycress), by optimizing the light intensity, the total osmotic pressure and the substrate composition of the liquid medium (Schwender and Ohlrogge, 2002; Lonien and Schwender, 2009; Chen and Shachar-Hill, 2012; Tsogtbaatar *et al.*, 2020; Sagun *et al.*, 2023). *In planta*, developing *Brassicaceae* embryos feed on the constituents of the surrounding endosperm liquid. Hence, determining the major organic components of the endosperm helps identifying the main source(s) of carbon and nitrogen for embryos (Schwender and Ohlrogge, 2002; Tsogtbaatar *et al.*, 2020).

Endosperm liquid was collected from 18-DAP *Physaria* seeds with an insulin syringe. To check the action of plant invertases, which cleave sucrose into fructose and glucose, uniformly ^13^C-labeled sucrose ([U-^13^C_12_]sucrose, *m*_+12_) was added to the liquid endosperm. Water-soluble metabolites were extracted from the collected endosperm, using boiling water to ensure the absence of enzymatic activities, and analysed by LC-MS/MS. Glucose and fructose were found to be the main sugars present in the liquid endosperm surrounding the embryo with a concentration of 69.7 ± 8.4 and 50.1 ± 6.0 mM, respectively (Supplementary Table S1). The [U-^13^C_12_]sucrose, added at the time of the collection, was not cleaved into hexoses, showing that the hexoses measured in the extract were actually present in the liquid endosperm (Supplementary Fig. S1). The main amino acids present in the liquid endosperm were found to be from the glutamine/glutamate family (i.e. Gln, Glu, and Pro) with a total concentration of 8.3 ± 2.0 mM (Supplementary Table S1). Abscisic acid (ABA) and salicylic acid were the most abundant hormones in *Physaria* endosperm at 2.1 ± 0.5 and 23.4 ± 8.8 µM, respectively (Supplementary Table S1). Knowing that it influences embryo development and hinders early root formation (Lee *et al.*, 2010; Rai *et al.*, 2011; Cheng *et al.*, 2014), ABA was selected to be added to the culture medium. Consequently, different ranges of substrates and hormone were tested on developing *Physaria* embryos with 25–150 mM glucose, 2.5–40 mM glutamine, and 0–20 µM ABA.

**Fig. 1.**
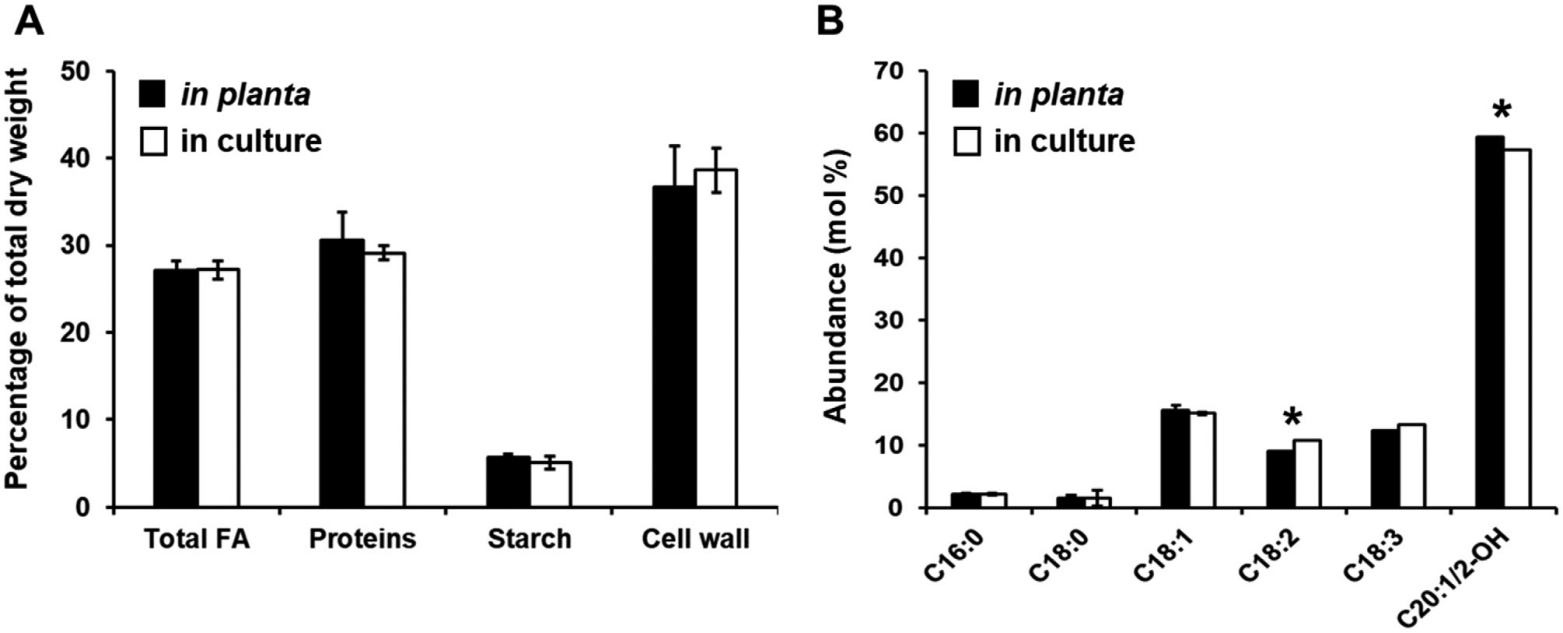
Validation of culture conditions for developing *Physaria* embryos. (A) Comparison of biomass composition from 27-DAP embryos *in planta* (black bars) and in culture embryos after 9 d of incubation (white bars). (B) Oil composition of *in planta* versus in culture embryos. The percentages of total dry weight and the abundance of each fatty acids from *Physaria* oil are the average and standard deviation of four biological replicates (*n*=4). *Statistically significant difference (*P*<0.05). C16:0, C18:0, C18:1, C18:2, C18:3, and C20:1/2-OH denote palmitic, stearic, oleic, linoleic, α-linolenic, and lesquerolic/auricolic acids, respectively.

To establish optimum osmotic pressure and light level for embryo cultures, several concentrations of polyethylene glycol (PEG 4000; 0–22.5%) and light intensities (5–50 μmol m^–2^ s^–1^) were assayed on *Physaria* embryos incubated with the substrates measured in the liquid endosperm. Physiological culture conditions were met when 18-DAP embryos were incubated for 9 d in 40 mM glucose, 5 mM glutamine, 10 µM ABA, 22.5% PEG with a light intensity of 12 μmol m^–2^ s^–1^. Indeed, in these conditions, (i) the growth rate of embryos after 9 d of culture (17.5 ± 2.1 µg embryo^−1^ d^−1^) was not significantly different from *in planta* at 27 DAP (15.4 ± 2.2 µg embryo^−1^ d^−1^; *P*=0.2), (ii) biomass composition was not significantly different (Fig. 1A), and (iii) the FA composition was very similar, with slightly but significantly more linoleic acid (C18:2) and less lesquerolic acid (C20:1-OH; Fig. 1B). These culture conditions, which mimic the development of *Physaria* embryos, can be used to determine the embryos’ carbon conversion efficiency and trace the main pathways that are active during oil biosynthesis using ^13^C-labeling.

### *Physaria* embryos convert carbon into biomass with a relatively modest efficiency

**Fig. 2.**
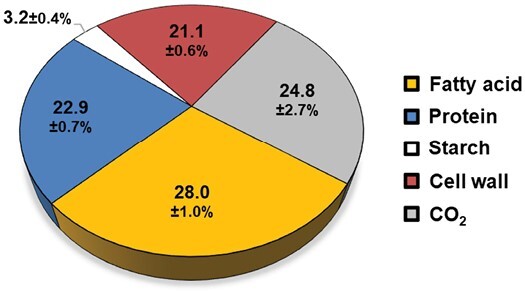
Distribution of carbon uptake by developing *Physaria* embryos. *Physaria* embryos store 75.2% of the carbon consumed from the media into biomass components: fatty acid (orange), protein (blue), starch (white), and cell wall (brown). The remaining 24.8% represents carbon released as CO_2_ (grey). Values are averages of four biological replicates ±SD.

*Brassicaceae* embryos synthesize biomass components using carbons received from the mother plant. The conversion of these carbons into oil results in a loss of CO_2_, and is therefore costly. Indeed, acetyl-CoA is the carbon precursor for *de novo* FAS. It is synthesized in the plastid through the pyruvate dehydrogenase complex. This complex catalyses the oxidative decarboxylation of pyruvate, generating acetyl-CoA and CO_2_. Therefore, for each two-carbon unit added to the nascent acyl-CoA chain, one carbon is lost as CO_2_, which makes the process of oil biosynthesis less efficient in comparison with other macromolecules (Goffman *et al.*, 2005). The efficiency by which *Physaria* embryos convert substrates into biomass (carbon conversion efficiency, CCE) was determined in developing embryos in culture for 9 d by measuring the substrates depleted from the medium and the biomass components produced during the incubation period (Fig. 2). The total carbon consumed from the medium was found to be 9.10 ± 0.26 µmol C per embryo of which 8.08 ± 0.23 and 1.02 ± 0.04 µmol C were from glucose and glutamine, respectively. In parallel, the total carbon stored in biomass components was 6.84 ± 0.02 µmol C per embryo, consisting of 2.55 ± 0.09, 2.09 ± 0.06, 0.29 ± 0.04, and 1.92 ± 0.05 µmol C in lipids, proteins, starch, and cell wall, respectively. Finally, the efficiency by which *Physaria* embryos convert carbon into biomass was estimated to be 75.2 ± 0.6% (Fig. 2), which is significantly lower than other photosynthetic embryos such as 82% for soybean (Allen *et al.*, 2009), 86% for rapeseed (Goffman *et al.*, 2005), and 93% for pennycress (Tsogtbaatar *et al.*, 2020). These findings indicate that there is potential to improve the overall carbon flow into oil in *Physaria*.

### Parallel ^13^C-labeling experiments and establishment of isotopic steady state

In order to determine the relative contribution of each metabolic pathway in terms of carbon skeletons, energy, and reductant for oil synthesis in *Physaria*, the substrates in the liquid medium were replaced with ^13^C-labeled ones, and parallel labeling experiments were conducted using: (i) 20% [U-^13^C_6_]glucose+20% [U-^13^C_5_]glutamine to check the isotopic steady state; (ii) 20% [U-^13^C_6_]glucose+80% [1,2-^13^C_2_]glucose and unlabeled glutamine; and (iii) unlabeled glucose and 100% [U-^13^C_5_]glutamine. Parallel labeling experiments (ii, iii) are designed to provide complementary information on the biochemical pathways active during oil synthesis (Schwender *et al.*, 2006; Alonso *et al.*, 2007, 2010a, 2011; Cocuron *et al.*, 2019b; Acket *et al.*, 2020; Tsogtbaatar *et al.*, 2020).

Isotopic steady state was validated by incubating 18-DAP *Physaria* embryos in 20% [U-^13^C_6_]glucose+20% [U-^13^C_5_]glutamine for 9 d. Then, intracellular metabolites and biomass components were extracted and their average carbon labeling was determined as described in ‘Material and methods’ (Tsogtbaatar *et al.*, 2020). Isotopic steady state was considered to be reached for compounds whose labeling abundance per carbon was in the range 18.0–23.0%. The results concluded that 32 out of the 39 analysed compounds reached the isotopic steady state (Supplementary Table S2). Glutamine, serine, and threonine were not significantly above the 23.0% limit, so they were included. The labeling abundance per carbon was found to be significantly lower than 18.0% for seven metabolites (glutamate, glycine, leucine, malate, glucose 6-P, glucose, and the cytosolic acetyl-CoA). Glucose and glutamate, with labeling abundances of 12.6 ± 1.0% and 6.8 ± 0.8%, did not reach the isotopic steady state and were, consequently, excluded. To be considered for further labeling interpretation, a dilution factor was applied to metabolites with labeling abundances between 16.0% and 18.0% (glycine, leucine, malate, glucose 6-P, and cytosolic acetyl-CoA) as previously described (Cocuron *et al.*, 2019b).

### Labeling with ^13^C-glucose highlights the operation of the oxidative pentose-phosphate pathway and fructose 1,6-bisphosphate aldolase

**Fig. 3.**
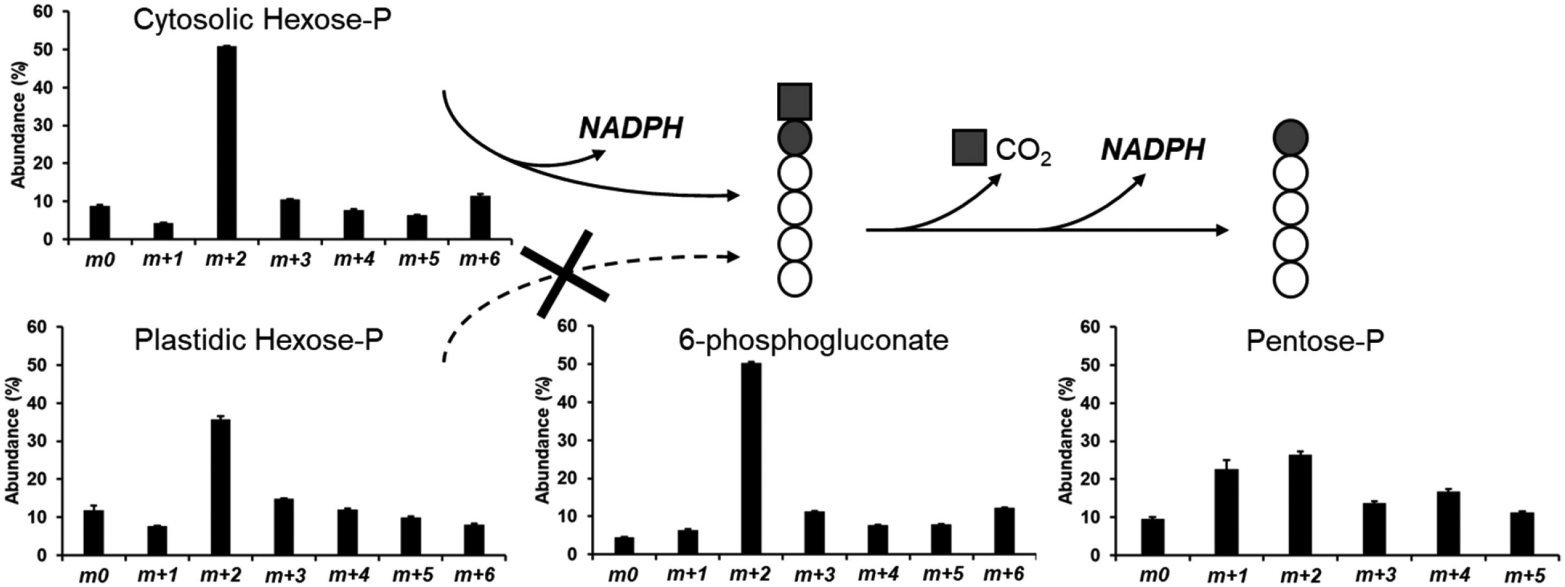
Occurrence and subcellular localization of the oxidative part of the OPPP. *Physaria* embryos were labeled with 20% [U-^13^C_6_]glucose+80% [1,2-^13^C_2_]glucose. The mass isotopomer distribution (MID) of each metabolite was determined by LC-MS/MS. Bar graphs are average of four biological replicates ±SD. MID of 6-phosphogluconate was compared with MIDs of pentose-P, cytosolic hexose-P obtained after sucrose hydrolysis (Cocuron *et al.*, 2020), and plastidic hexose-P obtained after starch hydrolysis. The dark square and circle follow the path of 6-phosphogluconate carbons 1 and 2, respectively.

After labeling *Physaria* embryos with 20% [U-^13^C_6_]glucose+80% [1,2-^13^C_2_]glucose, the MID and the labeling abundance per carbon was determined for each metabolite by LC-MS/MS (Supplementary Table S3). The occurrence and subcellular localization of the OPPP, an important pathway providing NADPH for biosynthesis, were investigated (Fig. 3). Feeding the embryos with 20% [U-^13^C_6_]glucose+80% [1,2-^13^C_2_]glucose resulted in a large abundance of 6-phophogluconate molecules containing two labeled carbons (*m*+2; Fig. 3). During the oxidative part of the OPPP, the first carbon of 6-phosphogluconate (six carbons) is lost as CO_2_, generating pentose-Ps (five carbons). The abundance pentose-P molecules with two labeled carbons (*m*+2) decreased in comparison with 6-phosphogluconate whereas molecules with one labeled carbon (*m*+1) increased, revealing that the OPPP is active during FAS in *Physaria* embryos (Fig. 3). In order to determine 6-phosphogluconate’s subcellular localization, its MID was compared with its potential precursors (hexose-Ps from the cytosol or plastid). To achieve this objective, enzymatic cleavage of sucrose and starch—exclusively synthesized in the cytosol and the plastid, respectively—into their hexose monomers was performed to uncover the labeling patterns of cytosolic and plastidic hexose-phosphates, respectively (Cocuron *et al.*, 2020). The MID of 6-phosphogluconate was found to be similar to those of cytosolic hexose-Ps (Fig. 3). Our results indicate that not only is the OPPP occurring in *Physaria* embryos, but the oxidative reactions are mostly active in the cytosol too, producing cytosolic NADPH, which may be used for FA elongation (Fig. 3).

**Fig. 4.**
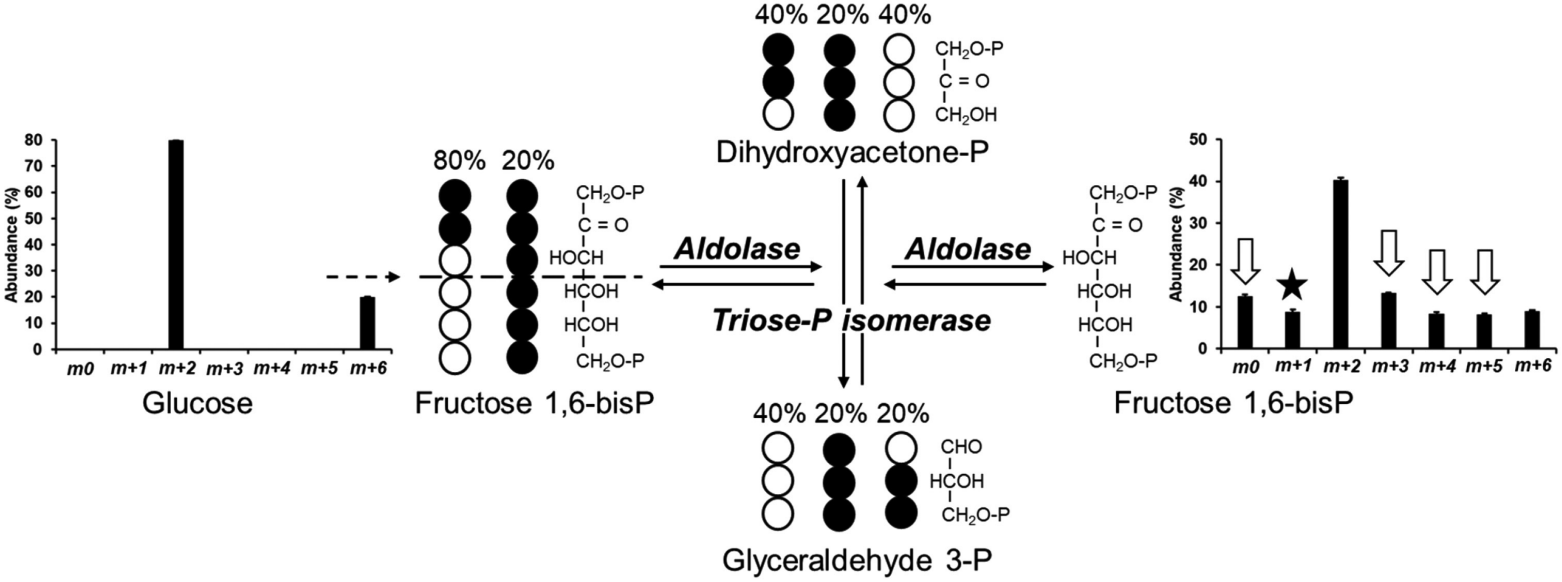
Randomization of the ^13^C-labeling during the reaction catalysed by the triose-P isomerase. *Physaria* embryos were labeled with 20% [U-^13^C_6_]glucose+80% [1,2-^13^C_2_]glucose. The mass isotopomer distribution (MID) of the fructose 1,6-bisP was determined by LC-MS/MS. Bar graphs are average of four biological replicates ±SD. Black and white circles follow the path of labeled and unlabeled carbon atoms, respectively. White arrows mark new mass isotopomers generated through the combined reactions of the aldolase/triose-P isomerase. The black star highlights a new mass isotopomer *m*+1 that comes from the oxidative part of the OPPP, as previously explained (Fig. 3).

The reversibility of the fructose 1,6-bisP aldolase (EC 4.1.2.13), catalysing the cleavage of fructose 1,6-bisP into two triose-Ps, and the occurrence of triose-P isomerase (EC 5.3.1.1) were investigated. After labeling *Physaria* embryos with 20% [U-^13^C_6_]glucose+80% [1,2-^13^C_2_]glucose, the theoretical MID for fructose 1,6-bisP would be approximately 80% and 20% for *m*+2 and *m*+6, respectively (Fig. 4). However, results showed abundance in all mass isotopomers (Fig. 4, Supplementary Table S2). The aldolase cleaves fructose 1,6-bisP into two triose-Ps: the top half of the fructose 1,6-bisP (carbons 1–3) yields dihydroxyacetone-P whereas the bottom half (carbons 4–6) yields glyceraldehyde 3-P. The isomerase randomizes the labeling between the two triose-Ps. Consequently, carbons 1 and 6 of the fructose 1,6-bisP end up as the third carbon of the glyceraldehyde 3-P, carbons 2 and 5 at the second position, and carbons 3 and 4 on the first position. After randomization of the labeling, the reversibility of the aldolase resynthesizes fructose 1,6-bisP from the triose-Ps, yielding new mass isotopomers *m*0, *m*+3, *m*+4, and *m*+6 (indicated by the white arrows, Fig. 4). No *m*+1 is generated through the combined reactions of the aldolase/triose-P isomerase. Instead *m*+1 measured in the fructose 1,6-bisP (highlighted by the black star, Fig. 4) comes from the oxidative part of the OPPP, as previously explained (Fig. 3).

### Labeling with ^13^C-glutamine reveals the occurrence of non-conventional pathways

**Fig. 5.**
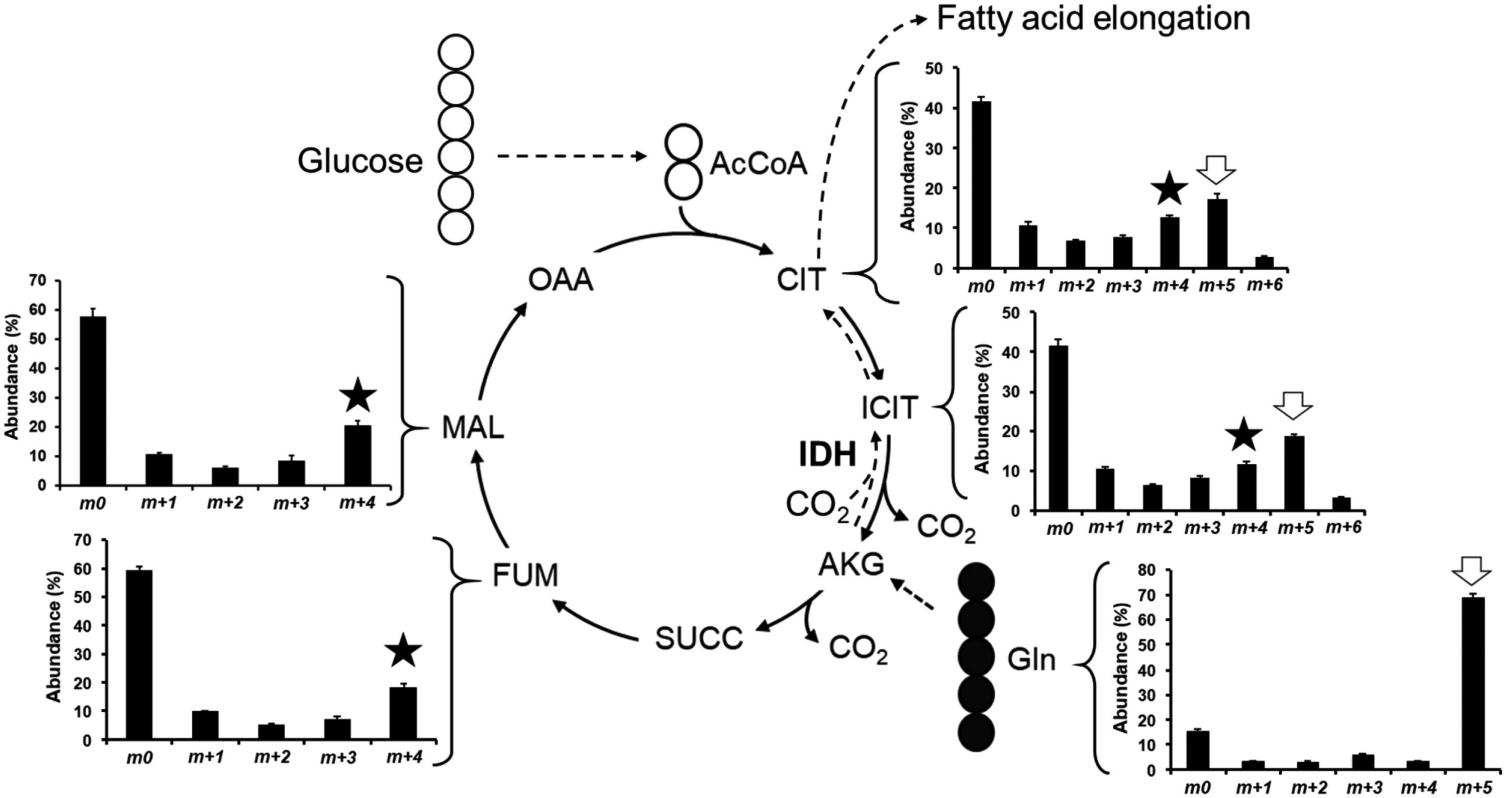
Reversibility of isocitrate dehydrogenase in developing *Physaria* embryos. *Physaria* embryos were labeled with 100% [U-^13^C_5_]glutamine. The mass isotopomer distribution (MID) of each metabolite was determined by LC-MS/MS. Bar graphs are average of four biological replicates ±SD. Black and white circles follow the path of labeled and unlabeled carbon atoms, respectively. The black star and the white arrow highlight the mass isotopomers *m*+4 and *m*+5, respectively. Figure adapted from Tsogtbaatar *et al.* (2020). Abbreviations: AcCoA, acetyl-coenzyme A; AKG, α-ketoglutarate; CIT, citrate; FUM, fumarate; ICIT, isocitrate; IDH, isocitrate dehydrogenase; MAL, malate; OAA, oxaloacetic acid; SUCC, succinate.

In parallel, *Physaria* embryos were labeled with 100% [U-^13^C_5_]glutamine, and the MIDs and average labeling per carbon (percent) in intracellular compounds was determined (Supplementary Table S3). Labeling in metabolites from the upper part of central metabolism, such as cytosolic glucose 6-P and fructose 6-P (obtained from sucrose hydrolysis), plastidic hexose-P (from starch hydrolysis), and pentose-Ps, was not significantly different from carbon’s natural abundance (1.1%), underlying the absence of gluconeogenesis. Regarding the TCA cycle, the most abundant labeled mass isotopomer in fumarate and malate was found to be *m*+4 (Fig. 5). It is due to the entry of ^13^C-labeled glutamine (Gln; 5 carbons) at the level of the α-ketoglutarate (five carbons), which gets decarboxylated into succinyl-CoA to form fumarate and malate (four carbons). This block of four ^13^C-carbons coming from glutamine and going through the traditional TCA cycle explains the high abundance of *m*+4 in these organic acids. Each cycle, a new molecule of unlabeled acetyl-CoA (AcCoA; two carbons) is condensed with an oxaloacetic acid (OAA; four carbons), resulting in the production of citrate and then isocitrate (six carbons). Interestingly, for citrate and isocitrate, the most abundant labeled mass isotopomer was found to be *m*+5 instead of *m*+4 (Fig. 5). This is due to the unusual and thermodynamically unfavorable reversibility of isocitrate dehydrogenase (IDH), which here catalyses the carboxylation of α-ketoglutarate (five carbons) into isocitrate (six carbons) too. This block of five ^13^C-carbons coming from glutamine and going through this unconventional pathway explains the higher abundance of *m*+5 in isocitrate and citrate. Interestingly, this unusual mechanism has been shown to operate in other photosynthetic embryos (Table 1). This reversibility of IDH not only leads to the capture of CO_2_, but also to the production of citrate to sustain FA elongation (Fig. 5).

**Fig. 6.**
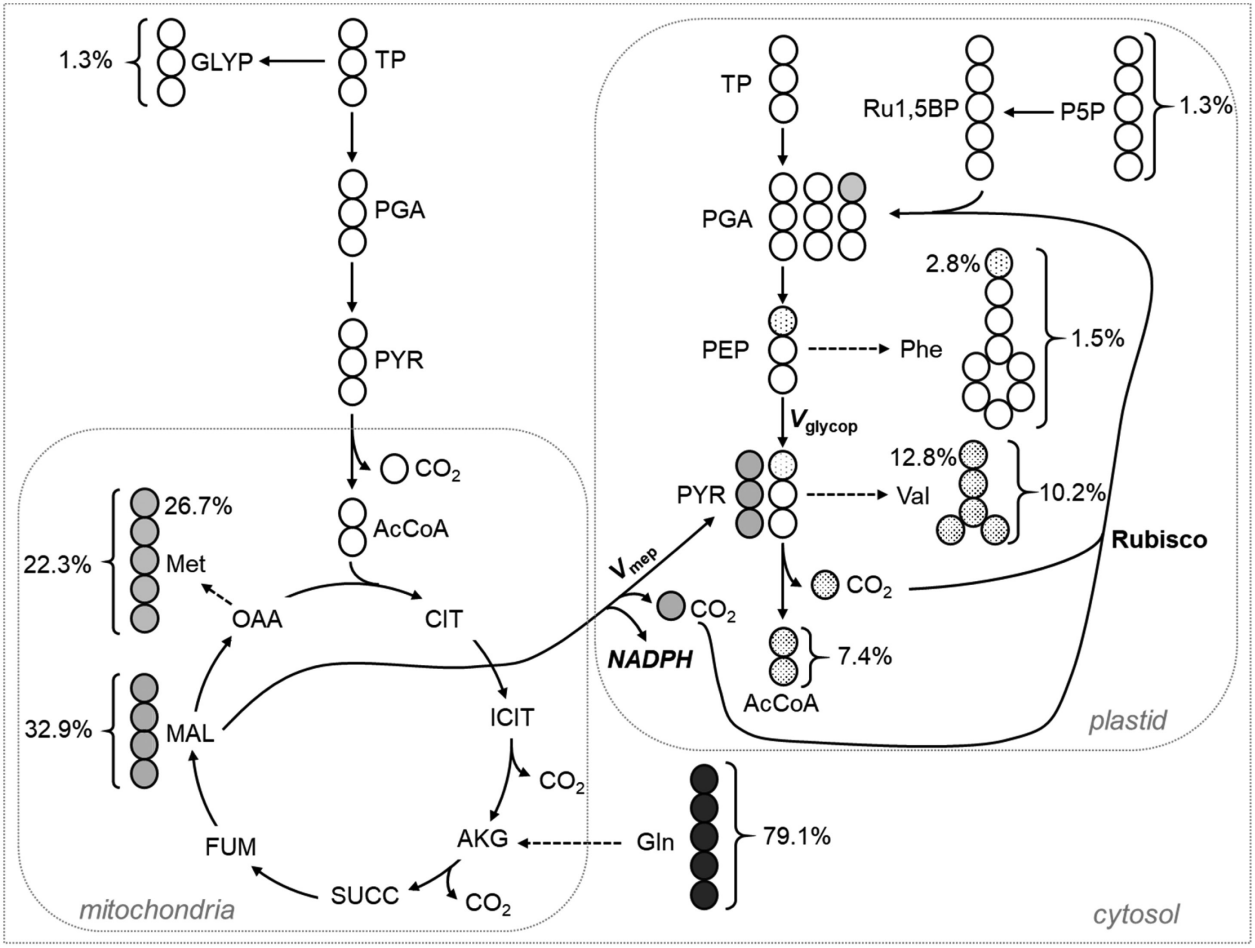
Operation of plastidic NADP-dependent malic enzyme and Rubisco in developing *Physaria* embryos. *Physaria* embryos were labeled with 100% [U-^13^C_5_]glutamine. The labeling of each metabolite was determined by LC-MS/MS. Grey and white circles follow the path of labeled and unlabeled carbon atoms, respectively. The shades of grey are proportional to the labeling. Values next to carboxylic groups are the ^13^C-enrichment (%) for the C1 of the molecules, and values next to brackets are the average labeling per carbon (%) of the molecule. The carboxyl groups of phenylalanine, valine, and methionine are derived from the ones of phospho*enol*pyruvate, pyruvate, and malate, respectively. Figure adapted from Tsogtbaatar *et al.* (2020). Abbreviations: AcCoA, acetyl coenzyme A; AKG, α-ketoglutarate; CIT, citrate; FUM, fumarate; GLYP, glycerol phosphate; ICIT, isocitrate; MAL, malate; OAA, oxaloacetate; P5P, pentose 5-phosphate; PGA, phosphoglycerate; PYR, pyruvate; Ru1,5BP, ribulose 1,5-bisphosphate; SUCC, succinate; TP, triose phosphates; *V*_glycop_, portion of pyruvate produced from plastidic glycolysis; *V*_mep_, portion of pyruvate produced by the plastidic NADP-dependent malic enzyme.

After labeling *Physaria* embryos with 100% [U-^13^C_5_]glutamine, significant labeling started to be detected in glycolysis at the level of PGA and PEP with 1.72 ± 0.13% and 1.74 ± 0.12%, respectively (Supplementary Table S3). The activity of the PEP carboxykinase (EC 4.1.1.32) would have converted four-carbon labeled molecules of OAA into three-carbon labeled PEP. Interestingly, the abundance of *m*+3 in PEP and PGA was found to be less than 0.5%, ruling out the contribution of PEP carboxykinase in developing *Physaria* embryos. However, the *m*+1 was the most abundant labeled isotopomer for PEP and PGA, which suggests the activity of Rubisco (Fig. 6), as explained step by step. First, valine, synthesized from plastidic pyruvate, had an average labeling per carbon significantly higher than the labeling enrichment of PEP (10.2% *vs* 1.7%, respectively). This difference is due to the decarboxylation of malate transported in the plastid into pyruvate by the activity of the NADP-dependent malic enzyme (Fig. 6). Using Equation 7, our labeling data showed that 41.8% of the plastidic pyruvate is produced by NADP-malic enzyme in *Physaria* embryos, which is the highest measured so far in developing photosynthetic embryos (Table 1). Therefore, the NADP-dependent malic enzyme highly contributes to channeling carbon and producing NADPH in the plastid for *de novo* FAS. Second, the operation of both decarboxylation reactions, those catalysed by NADP-dependent malic enzyme and pyruvate dehydrogenase, release substantial labeled CO_2_ inside the plastid (Fig. 6). The labeling enrichment of plastidic CO_2_ was estimated to be 24.0% utilizing Equation 8. Finally, our labeling data indicate that a large proportion of this CO_2_ is re-fixed by Rubisco (the Rubisco shunt) in developing *Physaria* embryos (Fig. 6). Indeed, the percentage of enrichment of ^13^C-carbon 1 from Val and Phe were determined to be at 12.8% and 2.8%, respectively (Fig. 6), which is significantly higher than carbon-13’s natural abundance. This labeling on carbon 1 of Val and Phe reflects the labeling of their direct precursor, i.e. the carboxylic group of plastidic PEP and pyruvate, respectively (Fig. 6). Therefore, the labeled CO_2_ released inside the plastid by the operation of NADP-dependent malic enzyme and pyruvate dehydrogenase is recaptured by Rubisco, leading to the labeling of carbon 1 of PEP and pyruvate. According to our labeling data, and using Equation 9, Rubisco contributes to the production of approximately 25% of the phosphoglycerate in developing *Physaria* embryos (Table 1).

In previous studies, a ratio of carbon going into oil to carbon released as CO_2_ above 2 was used as an indicator of the occurrence of unconventional pathways (Schwender *et al.*, 2004; Goffman *et al.*, 2005; Tsogtbaatar *et al.*, 2020). Indeed, acetyl-CoA—the two-carbon precursor for *de novo* FAS—is generated via the decarboxylation of pyruvate (three carbons) in the plastid. Therefore, for each three-carbon unit going to FAS, one is lost as a CO_2_, which leads to a ratio of carbon going into oil to carbon released as CO_2_ with maximal value of 2:1, knowing that the OPPP and the TCA cycle—pathways producing CO_2_—would decrease this ratio. A ratio above 2 reveals the operation of unusual pathways, such as the reversibility of IDH and Rubisco, which recapture internal CO_2_ released by other biochemical reactions (Schwender *et al.*, 2004). Although this ratio was found to be 1.1 (i.e. 2.55 µmol C into lipids/2.25 µmol C into CO_2_) in developing *Physaria* embryos, the results from the ^13^C-labeling highlighted the occurrence of non-conventional pathways (Figs 5, 6; Table 1). Similarly, in *Camelina sativa* embryos, the ratio of carbon going into oil to carbon released as CO_2_ was determined to be 0.6 due to (i) a high activity of OPPP producing large intracellular amounts of CO_2_, and (ii) no occurrence of Rubisco bypass (Carey *et al.*, 2020).

Similarly, ^13^C-labeling was conducted on developing embryos from other oilseed species (Table 1) (Schwender *et al.*, 2004, 2006; Alonso *et al.*, 2007, 2010a; Allen *et al.*, 2009; Hay *et al.*, 2014; Tsogtbaatar *et al.*, 2015; Cocuron *et al.*, 2019b; Acket *et al.*, 2020; Carey *et al.*, 2020). Interestingly, the non-canonical reversibility of IDH only occurred in ‘green’ embryos, which may be photosynthetically active. This reaction is thought to be important to generate citrate that is then cleaved in the cytosol by citrate lyase into oxaloacetate and acetyl-CoA to support FA elongation in oilseeds, which is certainly the case for rapeseed, camelina, pennycress, and *Physaria*. However, some species, such as flax, soybean, sunflower, and maize, do not have acyl chains above 18-carbon length; FAs of 18 carbons or fewer are synthesized in the plastid, and elongation is inactive. For developing maize and sunflower embryos—not photosynthetically active—the IDH reversibility does not occur whereas it is active for the ‘green’ embryos from flax and soybean. It is highly probable that there is a high demand of cytosolic acetyl-CoA generated from citrate for the production of secondary compounds, such as flavonoid biosynthesis, in flax and soybean (Oomah *et al.*, 1996; Malencic *et al.*, 2012; Chen *et al.*, 2023). Regarding the proportion of plastidic pyruvate produced from NADP-dependent malic enzyme (*V*_mep_), it varies from less than 1% for flax and rapeseed to up to 54% in maize (Table 1). A recent study in stable transgenic soybean expressing the plastid localized NADPH-dependent malic enzyme AtME4 (AT1G79750) showed an increase in oil content of 2–9% in mature seeds (Morley *et al.*, 2023). Developing *Physaria* embryos have the highest *V*_mep_ contribution measured so far in ‘green’ embryos, which highlights the particular importance of this pathway in providing not only carbon but also NADPH for *de novo* FAS. Finally, the recapture of CO_2_ by Rubisco (the Rubisco shunt) occurs in some of the ‘green’ embryos, and its contribution to plastidic PGA can reach up to 64% in rapeseed (Table 1). With 25%, Rubisco activity in developing *Physaria* embryos is similar to the one measured in pennycress. The operation of these non-conventional pathways—IDH, plastidic NADP-malic enzyme, and Rubisco—is supported by the transcript levels of genes encoding these enzymes in developing *Physaria* embryos (Horn *et al.*, 2016).

In conclusion, *Physaria* is a member of the *Brassicaceae* that produces a type of oil naturally rich in HFAs that is suitable for industrial applications. However, for this plant to be economically viable, its seed oil content must be improved. This study aims to gain a better understanding of the pathways involved in producing carbon for FAS in developing *Physaria* embryos in order to guide future metabolic engineering and/or breeding approaches. Based on the composition of the endosperm liquid, culture conditions that mimic the development of *Physaria* embryos were successfully established, which is key to determine the CCE and trace the main pathways involved in oil biosynthesis using ^13^C-labeling. This study demonstrated that *Physaria* embryos metabolize carbon into biomass with an efficiency significantly lower than other photosynthetic embryos. Interestingly, ^13^C-labeling revealed that *Physaria* uses non-conventional metabolic pathways to channel carbon into oil: (i) reversibility of isocitrate dehydrogenase, which provides additional carbon for FA elongation; (ii) plastidic NADP-dependent malic enzyme, which provides 42% of the pyruvate used for *de novo* FAS, which is the highest measured so far in developing ‘green’ oilseed embryos; and (iii) the Rubisco shunt, which fixes CO_2_ released in the plastid. We anticipate that metabolic engineering of these pathways will be key to improving carbon conversion efficiency and HFA content in *Physaria*.

## Supplementary data

The following supplementary data are available at *JXB* online.

Fig. S1. Identification and quantification of free sugars in the endosperm of *Physaria* seeds by LC-MS/MS.

Table S1. Sugar, amino acid, and hormone concentrations in *Physaria* endosperm.

Table S2. Metabolic isotopic steady state assessment by analysing the labeling abundance (%) per carbon of intracellular compounds from 20% ^13^C-labeling experiment.

Table S3. Determination of MID distribution and labeling abundance (%) per carbon of metabolites from [^13^C]glucose and [^13^C]glutamine parallel labeling experiments.

erad343_suppl_Supplementary_Figures_S1_Tables_S1-S3

## Data Availability

The data that support the findings are available within the paper and supporting data.

## Abbreviations

CCE: carbon conversion efficiency
DAP: days after pollination
DW: dry weight
FA: fatty acid
FAS: fatty acid synthesis
HFA: hydroxy fatty acid
IDH: isocitrate dehydrogenase
MID: mass isotopomer distribution
OPPP: oxidative pentose-phosphate pathway
PEP: phospho*enol*pyruvate
PGA: phosphoglycerate
TCA: tricarboxylic acid
