# Supplementary material for: 13C-labeling reveals non-conventional pathways providing carbon for hydroxy fatty acid synthesis in Physaria fendleri
Source: J Exp Bot. 2023 Sep 5;75(6):1754–66. doi: 10.1093/jxb/erad343 (PMC11275461; doi:10.1093/jxb/erad343)
Supplement: erad343_suppl_Supplementary_Figures_S1_Tables_S1-S3 [file erad343_suppl_supplementary_figures_s1_tables_s1-s3.pdf]

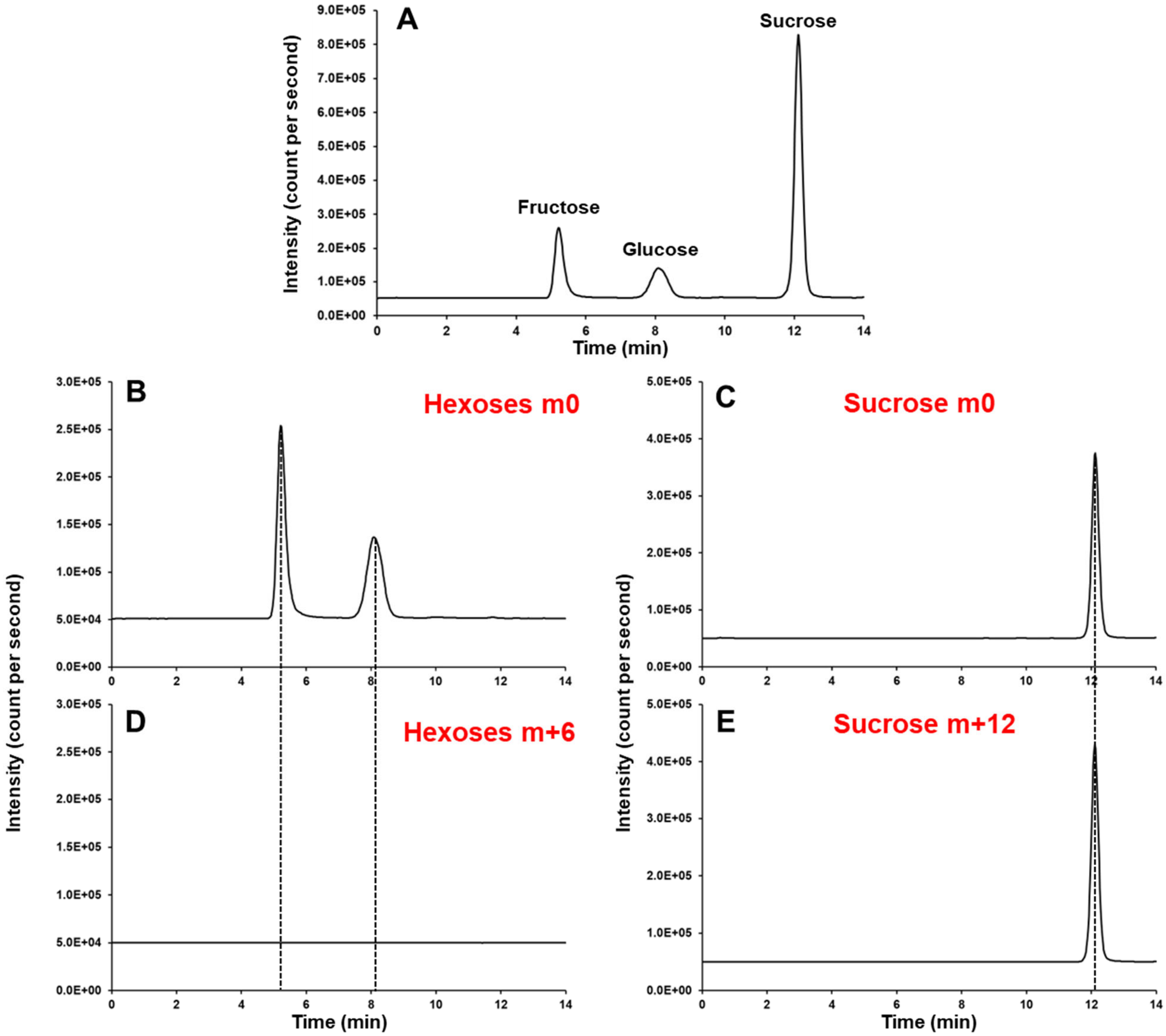

Fig. S1. Identification and quantification of free sugars in the endosperm of *Physaria* seeds by LC-MS/MS. Endosperm liquid was collected from 18 DAP seeds with an insulin syringe and [U-$^{13}C_{12}$]-sucrose was added. The extract was analyzed by LC-MS/MS using multiple reaction monitoring scan survey. (A) Total ion count: we followed sucrose unlabeled m0 (m/z = 340.9), [U-$^{13}C_{12}$]-sucrose m+12 (m/z = 352.9), unlabeled hexoses m0 (m/z = 178.9) and labeled hexoses m+6 (m/z = 184.9). Extracted ion chromatograms for (B) hexoses m0 (m/z = 178.9); (C) sucrose m0 (m/z = 340.9); (D) hexoses m+6 (m/z = 184.9); and (E) sucrose m+12 (m/z = 352.9).

***Table S1*. Sugar, amino acid, and hormone concentrations in *Physaria* endosperm.** Intracellular metabolites were detected and quantified using multiple reaction monitoring scan survey as indicated in the Material and Methods section. Analyte concentrations are the average ± standard deviation of four biological replicates (n=4). For sugars and amino acids, only compounds with average concentrations ≥ 1 mM are reported in this table.

Class of metabolites	Name	Concentration	
		Average	SD
Sugars (mM)	Glucose	69.7	8.4
	Fructose	50.1	6.0
	Sucrose	11.2	1.7
	Trehalose	1.0	0.3
Amino acids (mM)	Thr	4.5	1.0
	Gln	3.8	0.7
	Asp	2.5	0.4
	Glu	2.3	0.4
	Pro	2.2	0.9
	Val	1.9	0.3
	Ser	1.8	0.3
	Asn	1.1	0.1
Hormones (µM)	Salicylic acid	23.4	8.8
	Abscisic acid	2.1	0.5

***Table S2*. Metabolic isotopic steady state assessment by analyzing the labeling abundance (%) per carbon of intracellular compounds from 20 % ^{13}C-labeling experiment.** Metabolites with significant labeling enrichment above 23 % or under 18% are highlighted in bold. Compounds with labeling abundance significantly higher than 23 % or lower than 16 % were excluded from further analysis. The ones with enrichment value between 16-18 % were corrected according to the percentage shown in this table (Cocuron *et al.*, 2019b). Each percentage value is the average ± standard deviation of four biological replicates (n=4).

Class of metabolites	Names	Labeling abundance (%) per carbon	
		Average	SD
Amino acids	Ala	22.7	0.7
	Arg	20.9	0.2
	Asn	21.3	0.4
	Asp	22.7	0.6
	Gln	23.2	0.2
	**Glu**	**6.8**	**0.8**
	**Gly**	**16.1**	**2.6**
	His	20.5	0.3
	Ile	19.4	0.4
	**Leu**	**17.1**	**0.4**
	Met	18.8	0.3
	Phe	19.1	0.6
	Ser	23.3	1.4
	Thr	23.0	0.0
	Tyr	19.8	0.5
	Val	22.4	0.2
Organic acids	Citrate	19.6	0.3
	Fumarate	19.7	0.2
	Isocitrate	19.3	0.2
	**Malate**	**17.7**	**0.2**
	Trans-aconitate	20.1	0.5
Phosphorylated compounds	2/3-Phosphoglycerate	22.0	0.1
	6-phosphogluconate	21.6	0.4
	Fructose 1,6-bisphosphate	21.3	0.4
	Fructose 6-phosphate	22.0	0.6
	**Glucose 6-phosphate**	**17.6**	**0.3**
	Glycerol phosphate	21.0	1.7
	Pentose 5-phosphateS	22.0	0.1
	Phosphoenolpyruvate	21.6	0.3
	Sedoheptulose 7-phosphate	21.3	0.1
	Sucrose 6-phosphate	20.8	0.3
	Trehalose 6-phosphate	20.5	0.9
Free sugars	**Glucose**	**12.6**	**1.0**
	Sucrose	22.2	0.5
Compartimentalized metabolites	Plastidic acetyl-CoA unit	19.6	0.5
	**Cytosolic acetyl-CoA unit**	**16.5**	**1.0**
	Starch glucosyl unit	20.1	1.6
	Sucrose glucosyl unit	21.3	0.5
	Sucrose fructosyl unit	20.8	0.2

***Table S3*. Determination of MID distribution and labeling abundance (%) per carbon of metabolites from [^{13}C]-glucose and [^{13}C]-glutamine parallel labeling experiments.** Each mass isotopomer was analyzed by LC-MS/MS in the positive (amino acids) or negative mode (organic acids, phosphorylated compounds, free sugars, starch glucosyl units, sucrose fructosyl and glucosyl moieties). Labeling (%) per carbon of carboxyl fragments (highlighted in grey) obtained from LC-MS/MS is reported in the table as "C1" or "C6" for the corresponding amino acid. Determination of the labeling of plastidic and cytosolic hexoses was performed through starch glucosyl units and sucrose fructosyl and glucosyl moities, respectively (Cocuron *et al.*, 2020). GC-MS analysis was conducted to elucidate the labeling of plastidic and cytosolic acetyl-CoAs through McLafferty fragments of 18 carbon acyl chains and lesquerolic acid (C20:1-OH), respectively. Metabolites whose MID values were corrected by a dilution factor calculated as previously described (Cocuron *et al.*, 2019b) from the 20 % labeling experiment (Supplemental Table S1) are depicted with an asterisk (*). Each percentage value is the average ± standard deviation of four biological replicates (n=4).

Metabolites	Mass isotopomers	Labeling with [^{13}C]-Glucose				Labeling with [^{13}C]-Glutamine			
		% ^{13}C abundance		Labeling (%) per carbon		% ^{13}C abundance		Labeling (%) per carbon	
		Average	SD	Average	SD	Average	SD	Average	SD
Ala	M+0	41.15	0.50	42.88	1.51	85.59	0.54	9.17	0.29
	M+1	9.02	0.48			7.04	0.38		
	M+2	30.80	1.20			1.83	0.04		
	M+3	19.34	0.55			5.61	0.13		
C1-Ala				26.42	0.53			10.09	0.52
Arg	M+0	43.72	0.15	20.54	0.68	25.95	0.74	50.27	0.87
	M+1	22.74	0.60			9.68	0.04		
	M+2	14.18	0.47			4.58	0.05		
	M+3	9.71	0.18			10.13	0.26		
	M+4	5.91	0.37			3.77	0.10		
	M+5	3.18	0.08			39.86	0.34		
	M+6	0.58	0.02			6.34	0.36		
C6-Arg				27.84	0.47			14.95	0.26
Asn	M+0	41.54	0.83	32.11	1.13	56.26	2.88	31.70	2.97
	M+1	15.11	0.34			8.50	1.56		
	M+2	22.29	0.81			7.39	1.26		
	M+3	16.20	0.31			9.28	1.24		
	M+4	5.04	0.40			18.91	1.02		
Asp	M+0	40.30	0.55	32.99	0.84	55.99	1.63	29.50	2.88

	M+1	14.80	0.45			13.10	0.61		
	M+2	23.60	0.24			6.05	0.11		
	M+3	16.07	0.53			7.88	1.59		
	M+4	5.44	0.22			17.29	1.49		
Gln	M+0	71.53	0.69	13.36	0.41	14.48	0.94	79.06	1.58
	M+1	7.41	0.42			3.27	0.41		
	M+2	10.97	0.20			3.19	0.15		
	M+3	4.70	0.15			5.90	0.27		
	M+4	3.84	0.11			3.52	0.09		
	M+5	1.60	0.07			70.77	1.21		
Gly*	M+0	50.33	3.53	32.61	7.96	68.94	1.13	18.44	7.18
	M+1	34.69	9.10			25.57	6.31		
	M+2	15.27	3.41			5.65	4.03		
His	M+0	6.18	0.55	49.84	1.90	92.26	0.34	1.37	0.18
	M+1	12.19	0.42			7.51	0.11		
	M+2	21.67	0.47			0.07	0.09		
	M+3	22.15	0.25			0.04	0.06		
	M+4	17.72	0.70			0.05	0.08		
	M+5	14.45	0.70			0.04	0.05		
	M+6	5.65	0.50			0.00	0.01		
C1-His				49.75	1.01			1.61	0.19
Ile	M+0	23.34	0.61	36.15	2.23	54.46	0.72	20.62	1.96
	M+1	9.33	1.15			13.28	0.38		
	M+2	29.61	1.38			7.11	0.44		
	M+3	14.82	1.08			6.87	1.51		
	M+4	13.65	0.95			16.77	0.86		
	M+5	6.57	0.41			0.60	0.25		
	M+6	2.73	0.06			0.92	0.21		
C1-Ile				23.48	0.79			26.38	1.22
Leu*	M+0	14.59	0.56	46.33	1.79	65.64	0.49	10.32	1.41
	M+1	8.03	1.86			13.69	0.55		
	M+2	26.86	0.66			16.27	0.71		
	M+3	9.23	0.30			2.43	0.46		

	M+4	27.60	0.36			1.54	0.77		
	M+5	4.72	0.76			0.35	0.35		
	M+6	9.09	0.24	-	-	0.08	0.05		
Met	M+0	25.95	1.11	32.79	1.11	58.49	1.54	22.31	2.40
	M+1	26.78	0.13			12.41	1.16		
	M+2	19.08	0.34			5.14	0.47		
	M+3	17.90	0.13			9.84	2.26		
	M+4	8.92	1.04			14.76	0.77		
	M+5	1.92	0.04			0.06	0.02		
C1-Met				23.91	1.20			26.70	1.80
Phe	M+0	11.84	0.61	41.16	1.03	87.42	0.48	1.55	0.15
	M+1	5.20	0.04			11.79	0.48		
	M+2	14.35	0.20			0.54	0.04		
	M+3	14.89	0.55			0.16	0.07		
	M+4	16.33	0.35			0.02	0.01		
	M+5	15.26	0.27			0.03	0.03		
	M+6	10.88	0.27			0.02	0.02		
	M+7	6.29	0.20			0.02	0.02		
	M+8	3.87	0.13			0.01	0.01		
	M+9	1.10	0.04			0.00	0.00		
C1-Phe				24.71	0.19			2.77	0.19
Ser *	M+0	36.60	2.74	41.67	13.68	89.26	1.87	7.30	5.40
	M+1	19.03	2.99			4.11	2.68		
	M+2	28.25	4.12			2.32	0.71		
	M+3	16.49	9.93			4.38	4.03		
Thr *	M+0	41.59	1.17	32.27	2.34	53.85	53.85	32.19	32.19
	M+1	14.64	1.08			12.12	12.12		
	M+2	22.71	1.80			6.09	6.09		
	M+3	15.85	0.55			8.47	8.47		
	M+4	5.37	0.76			19.76	19.76		
Tyr	M+0	9.20	1.24	42.29	2.23	85.14	0.90	2.83	1.22
	M+1	5.47	0.58			12.14	0.64		
	M+2	15.57	0.81			0.47	0.08		

	M+3	14.33	0.35			0.20	0.09		
	M+4	17.21	0.55			0.18	0.17		
	M+5	15.69	1.09			1.18	0.93		
	M+6	10.83	0.22			0.08	0.14		
	M+7	6.49	0.56			0.21	0.20		
	M+8	4.05	0.38			0.40	0.28		
	M+9	1.21	0.10			0.00	0.00		
C1-Tyr				25.43	0.92			2.79	0.16
Val	M+0	18.01	0.11	46.44	0.83	74.85	1.44	10.16	0.68
	M+1	7.20	0.23			9.23	0.52		
	M+2	34.40	0.63			7.55	0.44		
	M+3	15.07	0.05			7.39	0.45		
	M+4	16.36	0.57			0.63	0.13		
	M+5	9.11	0.05			0.35	0.03		
C1-Val				27.31	0.22			12.78	0.76
Citrate	M+0	32.23	2.13	31.01	2.12	41.65	1.26	33.97	1.93
	M+1	14.86	0.80			10.84	0.79		
	M+2	25.25	1.98			6.86	0.23		
	M+3	12.95	0.78			7.92	0.46		
	M+4	10.97	0.77			12.67	0.60		
	M+5	5.50	0.39			17.38	1.09		
	M+6	1.74	0.10			2.99	0.19		
Fumarate	M+0	55.81	8.98	33.81	7.24	59.46	1.35	28.81	2.35
	M+1	15.25	3.10			10.03	0.20		
	M+2	24.04	5.10			5.34	0.43		
	M+3	16.04	3.63			7.15	0.92		
	M+4	5.95	1.19			18.27	1.39		
Isocitrate	M+0	35.21	2.96	29.75	2.60	41.68	1.39	34.56	1.56
	M+1	15.04	1.04			10.44	0.64		
	M+2	24.13	2.66			6.45	0.12		
	M+3	12.09	1.10			8.20	0.44		
	M+4	10.68	0.60			11.61	0.77		
	M+5	5.29	0.61			18.65	0.72		

	M+6	1.62	0.08			3.29	0.08		
Malate*	M+0	46.00	1.86	32.42	1.34	57.79	2.64	32.92	3.17
	M+1	13.90	0.70			10.86	0.44		
	M+2	23.11	1.29			6.18	0.48		
	M+3	15.66	0.50			8.64	1.76		
	M+4	5.64	0.15			20.63	1.50		
Trans-aconitate	M+0	34.43	2.26	28.20	1.11	42.78	1.39	33.21	1.94
	M+1	14.30	0.61			10.71	0.60		
	M+2	23.39	0.95			6.75	0.25		
	M+3	11.95	0.52			7.78	0.20		
	M+4	9.38	0.41			12.47	0.84		
	M+5	5.10	0.16			16.80	0.91		
	M+6	1.54	0.02			2.97	0.34		
2/3-Phosphoglycerate	M+0	39.31	0.44	45.71	0.32	94.98	0.11	1.72	0.13
	M+1	7.32	0.41			5.08	0.17		
	M+2	32.82	0.20			0.00	0.04		
	M+3	21.39	0.05			0.20	0.04		
6-phosphogluconate	M+0	4.57	0.09	47.53	0.51	92.50	0.09	1.10	0.20
	M+1	6.41	0.32			8.31	0.36		
	M+2	50.27	0.27			0.00	0.39		
	M+3	11.28	0.20			0.00	0.02		
	M+4	7.68	0.12			0.00	0.01		
	M+5	7.98	0.12			0.00	0.00		
	M+6	12.29	0.09			0.00	0.00		
Fructose 1,6-bisphosphate	M+0	12.58	0.29	42.89	1.06	91.71	0.35	1.62	0.44
	M+1	8.83	0.44			7.97	0.17		
	M+2	40.25	0.61			0.00	0.06		
	M+3	13.35	0.07			0.03	0.24		
	M+4	8.33	0.43			0.09	0.14		
	M+5	8.14	0.25			0.09	0.09		
	M+6	9.00	0.25			0.15	0.10		
Fructose 6-phosphate	M+0	4.63	0.27	47.89	0.83	92.13	0.82	1.45	0.21

	M+1	5.99	0.27			7.81	0.58		
	M+2	48.48	0.39			0.00	0.20		
	M+3	12.52	0.19			0.01	0.02		
	M+4	8.82	0.34			0.01	0.01		
	M+5	8.39	0.20			0.20	0.03		
	M+6	11.60	0.16			0.03	0.00		
Glucose 6-phosphate*	M+0	6.55	0.58	47.18	0.73	83.63	0.22	2.41	0.20
	M+1	5.48	0.12			18.54	0.82		
	M+2	48.00	0.47			0.00	0.10		
	M+3	12.00	0.19			0.06	0.03		
	M+4	8.36	0.19			0.03	0.01		
	M+5	8.06	0.04			0.01	0.00		
	M+6	11.98	0.30			0.02	0.00		
Glycerol phosphate	M+0	38.66	1.57	45.85	1.53	95.86	0.16	1.26	0.09
	M+1	7.12	0.26			4.54	0.10		
	M+2	34.47	1.65			0.00	0.07		
	M+3	20.50	0.34			0.03	0.01		
Pentose 5-phosphateS	M+0	9.67	0.42	47.97	2.05	93.45	0.25	1.29	0.08
	M+1	22.73	2.24			6.74	0.22		
	M+2	26.40	0.87			0.00	0.04		
	M+3	13.72	0.50			0.03	0.01		
	M+4	16.78	0.73			0.02	0.01		
	M+5	11.21	0.37			0.00	0.01		
Phosphoenolpyruvate	M+0	38.15	1.01	46.75	1.31	95.20	0.11	1.74	0.12
	M+1	7.36	0.33			4.77	0.05		
	M+2	32.81	0.88			0.00	0.07		
	M+3	22.42	0.61			0.38	0.06		
Sedoheptulose 7-phosphate	M+0	2.69	0.30	51.04	1.04	90.62	0.45	1.36	0.10
	M+1	7.87	0.26			9.33	0.47		
	M+2	18.81	0.10			0.00	0.07		
	M+3	21.57	0.44			0.04	0.02		
	M+4	19.72	0.49			0.01	0.00		

	M+5	14.31	0.33			0.00	0.00		
	M+6	10.95	0.12			0.00	0.00		
	M+7	4.42	0.18			0.00	0.00		
Sucrose 6-phosphate	M+0	0.34	0.07	47.18	1.53	87.54	0.86	1.15	0.13
	M+1	0.33	0.02			11.59	0.77		
	M+2	5.20	0.51			0.70	0.20		
	M+3	4.70	0.14			0.06	0.02		
	M+4	31.84	1.16			0.02	0.01		
	M+5	12.23	0.22			0.03	0.01		
	M+6	10.28	0.27			0.03	0.02		
	M+7	10.09	0.10			0.01	0.01		
	M+8	16.17	0.22			0.00	0.00		
	M+9	3.48	0.37			0.00	0.00		
	M+10	2.03	0.24			0.00	0.00		
	M+11	1.64	0.08			0.00	0.00		
	M+12	1.78	0.03			0.00	0.00		
Trehalose 6-phosphate	M+0	0.20	0.01	46.80	4.14	87.08	0.50	1.25	0.15
	M+1	0.45	0.05			11.80	0.46		
	M+2	5.48	0.31			0.93	0.13		
	M+3	5.92	0.28			0.00	0.05		
	M+4	30.64	2.01			0.02	0.01		
	M+5	12.51	0.96			0.01	0.01		
	M+6	10.21	0.74			0.08	0.03		
	M+7	10.49	1.09			0.02	0.02		
	M+8	15.38	1.96			0.03	0.03		
	M+9	3.45	0.26			0.03	0.02		
	M+10	2.14	0.21			0.00	0.00		
	M+11	1.76	0.13			0.01	0.01		
	M+12	1.49	0.14			0.00	0.01		
Sucrose	M+0	0.32	0.06	47.28	1.45	82.55	0.92	1.55	0.06
	M+1	0.37	0.03			14.88	0.32		
	M+2	5.11	0.19			1.70	0.10		

	M+3	4.92	0.33			0.13	0.04		
	M+4	30.73	0.42			0.00	0.01		
	M+5	12.52	0.55			0.00	0.00		
	M+6	10.46	0.44			0.00	0.00		
	M+7	9.86	0.30			0.00	0.00		
	M+8	16.95	0.48			0.00	0.00		
	M+9	3.81	0.16			0.00	0.00		
	M+10	2.33	0.09			0.00	0.00		
	M+11	1.88	0.06			0.00	0.00		
	M+12	0.90	0.02			0.00	0.00		
Cytosolic acetyl-CoA unit	M+0	55.90	0.48	37.79	0.78	63.18	0.38	31.70	0.87
	M+1	15.68	0.12			12.84	0.08		
	M+2	29.95	0.72			25.28	0.83		
Plastidic acetyl-CoA unit	M+0	48.82	0.16	47.13	0.12	89.62	0.10	7.36	0.11
	M+1	12.23	0.12			6.50	0.09		
	M+2	41.01	0.06			4.11	0.06		
Starch glucosyl unit (Plastidic hexose-Phosphates)	M+0	11.84	1.16	45.14	0.90	92.84	0.06	1.34	0.05
	M+1	7.62	0.12			6.32	0.04		
	M+2	35.73	0.77			0.81	0.05		
	M+3	14.88	0.14			0.03	0.03		
	M+4	12.00	0.28			0.00	0.01		
	M+5	10.05	0.18			0.00	0.01		
	M+6	8.14	0.21			0.00	0.00		
Sucrose fructosyl unit (Cytosolic fructose-6 Phosphate)	M+0	5.25	0.23	46.85	0.68	92.93	0.16	1.24	0.11
	M+1	3.71	0.12			6.75	0.26		
	M+2	54.82	0.16			0.29	0.12		
	M+3	10.56	0.33			0.05	0.06		
	M+4	6.57	0.15			0.00	0.00		
	M+5	6.68	0.16			0.00	0.00		
	M+6	12.73	0.21			0.00	0.00		
Sucrose glucosyl unit (Cytosolic glucose-6	M+0	8.81	0.31	45.01	0.69	92.73	0.23	1.35	0.15
	M+1	4.34	0.10			6.68	0.00		

Phosphate)	M+2	50.88	0.10	0.36	0.10
	M+3	10.60	0.06	0.15	0.06
	M+4	7.73	0.21	0.02	0.06
	M+5	6.38	0.12	0.03	0.06
	M+6	11.56	0.37	0.00	0.00
